# Present and Future of Immunotherapy in Patients With Glioblastoma: Limitations and Opportunities

**DOI:** 10.1093/oncolo/oyad321

**Published:** 2023-12-04

**Authors:** Marta Maccari, Chooyoung Baek, Mario Caccese, Susanna Mandruzzato, Alba Fiorentino, Valeria Internò, Alberto Bosio, Giulia Cerretti, Marta Padovan, Ahmed Idbaih, Giuseppe Lombardi

**Affiliations:** Department of Oncology, Oncology 1, Veneto Institute of Oncology IOV-IRCCS, Padova, Italy; Sorbonne Université, AP-HP, Institut du Cerveau - Paris Brain Institute - ICM, Inserm, CNRS, Hôpitaux Universitaires La Pitié Salpêtrière - Charles Foix, DMU Neurosciences, Service de Neurologie 2-Mazarin, Paris, France; Department of Oncology, Oncology 1, Veneto Institute of Oncology IOV-IRCCS, Padova, Italy; Department of Surgery, Oncology and Gastroenterology, University of Padova, Padova, Italy; Immunology and Molecular Oncology Diagnostics, Veneto Institute of Oncology IOV - IRCCS, Padova, Italy; Department of Radiation Oncology, Miulli General Regional Hospital, Acquaviva delle Fonti, Bari, Italy; Department of Medicine and Surgery, LUM University, Casamassima, Bari, Italy; Oncology Unit, San Paolo Hospital, Bari, Italy; Department of Oncology, Oncology 1, Veneto Institute of Oncology IOV-IRCCS, Padova, Italy; Department of Oncology, Oncology 1, Veneto Institute of Oncology IOV-IRCCS, Padova, Italy; Department of Oncology, Oncology 1, Veneto Institute of Oncology IOV-IRCCS, Padova, Italy; Sorbonne Université, AP-HP, Institut du Cerveau - Paris Brain Institute - ICM, Inserm, CNRS, Hôpitaux Universitaires La Pitié Salpêtrière - Charles Foix, DMU Neurosciences, Service de Neurologie 2-Mazarin, Paris, France; Department of Oncology, Oncology 1, Veneto Institute of Oncology IOV-IRCCS, Padova, Italy

**Keywords:** glioblastoma, immunotherapy, CAR T cell, checkpoint inhibitors, vaccines

## Abstract

Glioblastoma (GBM) is the most common and aggressive primary malignant brain tumor. Standard therapies, including surgical resection, chemoradiation, and tumor treating fields, have not resulted in major improvements in the survival outcomes of patients with GBM. The lack of effective strategies has led to an increasing interest in immunotherapic approaches, considering the success in other solid tumors. However, GBM is a highly immunosuppressive tumor, as documented by the presence of several mechanisms of immune escape, which may represent a reason why immunotherapy clinical trials failed in this kind of tumor. In this review, we examine the current landscape of immunotherapy strategies in GBM, focusing on the challenge of immunoresistance and potential mechanisms to overcome it. We discussed completed and ongoing clinical trials involving immune checkpoint inhibitors, oncolytic viruses, vaccines, and CAR T-cell therapies, to provide insights into the efficacy and outcomes of different immunotherapeutic interventions. We also explore the impact of radiotherapy on the immune system within the GBM microenvironment highlighting the complex interactions between radiation treatment and the immune response.

## Teaser

This review examines the current landscape of immunotherapy strategies in glioblastoma, focusing on the challenge of immunoresistance and potential mechanisms to overcome it.

**Implications for Practice**

The immunotherapy landscape is constantly expanding. Even though, to date, immunotherapy has not significantly impacted clinical practice in the treatment of glioblastoma, it is crucial for clinicians being up to date on the current “state of the art” of immunotherapy in GBM because it may represent a future direction for the treatment of this aggressive primary brain tumor.

## Introduction

Glioblastoma (GBM) is the most common and lethal primary brain cancer in adults.^1^ First-line therapy for newly diagnosed GBM is represented by maximal safe resection, followed by concomitant chemoradiotherapy and maintenance therapy with temozolomide.^2^ In subgroups of patients, prescription of lomustine and tumor treating fields provide additional clinical benefit^3,4^

Despite this aggressive standard of care (SoC), disease progression is inevitable, with 15-18 months of median overall survival (mOS) and a 5-year survival rate of <5%. For patients with unresectable tumor, the prognosis is even poorer.^5^

In recent years, the dismal prognosis of patients with GBM led to major research efforts to discover new therapeutical options.

Cancer immunosurveillance is the concept that immune system can actively identify and potentially eliminate cancer cells. However, some tumor cells develop the ability to evade the control of the immune system through a number of mechanisms (eg, loss or downregulation of tumor antigens and MHC (major histocompatibility complex) molecules, expression of inhibiting proteins, metabolic inhibition).^6^ Cancer immunotherapy focuses on overcoming immunoresistance of tumor cells to promote cancer eradication.

Based on this assumption, significant results in several types of solid and hematological tumors have been reached, especially through the development of immune checkpoint inhibitors and chimeric antigen receptor (CAR) T-cell therapy,^7-12^ thus generating a growing interest in the application of these therapeutical strategies for patients affected by GBM.

However, the initial enthusiasm has been mitigated by the recognition of the immunosuppressive nature of GBM.^13^ It is well known that GBM stands as a “cold tumor” that develops in a unique immune environment such as the central nervous system (CNS) and that sets in motion several mechanisms of immunosuppression.^14,15^ A better knowledge of these factors is needed for an effective implementation of immunotherapy in GBM.

**Figure 1.**
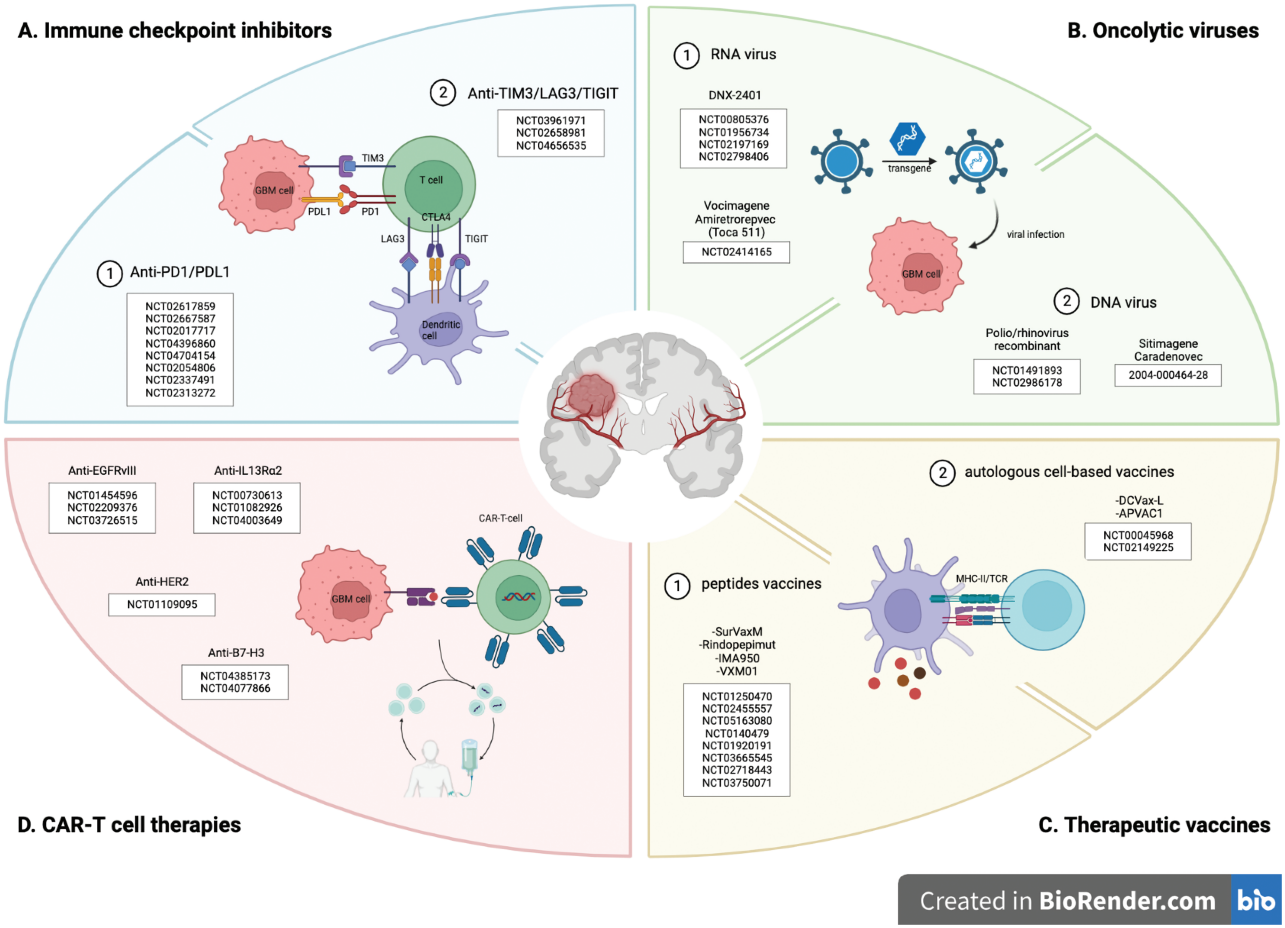
Overview of the current landscape of immunotherapy strategies in GBM and related clinical trials. **A**. Immune checkpoint inhibitors are specific antibodies that block PD1/PDL1 or CTLA4 pathways, and TIM3, LAG3, and TIGIT coinhibitory molecules. **B**. Oncolytic viruses are RNA or DNA viruses that selectively infect and kill cancer cells. **C**. Therapeutic vaccines are peptide or “autologous” cell-based vaccines that activate T cells to target tumor antigens. **D**. CAR T-cell therapies target GBM-associated or GBM-specific antigens including EGFRvIII, IL13Ra2, HER2, and B7-H3. Image created in BioRender.com.

Herein, we review ongoing and completed clinical and pre-clinical trials on immunotherapeutical approaches for GBM with a particular focus on immune checkpoint-inhibitors, tumor vaccines, and CAR T-cell therapies (Figure 1). We will also provide insight on mechanisms of resistance, and on the relationship between radiotherapy (RT) and immune system, the understanding of which is crucial for the development of novel immunotherapeutical strategies.

## Glioblastoma Immunoresistance and Possible Strategies to Overcome

Despite the blooming success of cancer immunotherapy in many tumor types, GBM stood out as a model of resistance to immune-mediated destruction. Several works studied the relationship between GBM and immune system and shed light on a number of mechanisms that restrain the activity of the immune system. In addition, it has to be considered that such mechanisms of immune suppression take place in the CNS, that is, in an anatomic location separated by a blood-brain barrier (BBB) that for decades was believed to be the first line of defense of the brain, actively excluding most peripheral immune cells. This feature, together with the finding that the non-syngeneic tissues were not rejected from the brain of laboratory animals, led to consider for a long time the CNS as an immune privileged site.^16^ Only recent advancement in the field of neuroanatomy enabled to revise the concept of immune privilege of the CNS and to introduce the idea of a flexible BBB and of a peculiar immune surveillance adapted to this site.^17^

Nowadays, a large body of evidence indicates that GBM immunosuppressive control extends over the boundaries of the CNS. As shown by Fecci et al^18^, one of the dysfunctions observed in patients with GBM is the T-cell lymphopenia in treatment-naïve patients due to the sequestration of T cells in the bone marrow regulated by the tumor imposed loss of S1P1 from the T-cell surface. Another mechanism of immune suppression is demonstrated by the presence in peripheral blood of an increased level of Arginase 1 (Arg1), led by neutrophils degranulation that exert an immune suppressive activity on T cells.^19,20^ Arg1 is an enzyme constitutively expressed in neutrophils stored within intracellular granules and we showed that its enzymatic activity is positively associated with glioma grade and represent an independent risk factor of disease progression and survival of patients with GBM.^20^ Of note, T-cell dysfunction can be restored by targeting Arg1 in vitro,^19^ thus showing another target of potential pharmacological intervention.

A key feature of GBM is the presence of an immune suppressive tumor microenvironment (TME), largely dominated by the influx of blood-derived macrophages endowed with a strong immune suppressive activity. Of note, in GBM, the BBB is largely compromised allowing the infiltration of several immune system cells from the peripheral circulation, but while blood-derived macrophages are present in large amount, very few cells of the adaptive immune system are present, thus showing that in this tumor there is a restricted access, facilitating the entrance of tumor-promoting cells, and excluding the presence of potential antitumor effector cells. A striking feature of the blood-derived macrophages is their acquisition of an immunosuppressive phenotype. In this respect, we demonstrated that blood-derived macrophages modulate their immune suppressive activity according to the location within the TME, with the highest activity in the central tumor area, and a lower activity in the marginal area.^21^ We also showed the presence of a significant iron metabolism in these macrophages, and that blocking this metabolic pathway can contribute to fight immune tolerance.^22^ Interestingly, Platten et al^23^ showed that in IDH (Isocitrate Dehydrogenase) -mutated tumors the differentiation of infiltrating myeloid cells is blocked and that tryptophan metabolism drives the macrophages into an immunosuppressive state. Remarkably, pharmacological inhibition of tryptophan metabolism can reverse immunosuppression, thus stressing the point that understanding these metabolic mechanisms is important, especially toward the goal of developing optimized new therapies.

The lack of T cells in the GBM parenchyma could be due to several factors, such as the low mutational burden, the low number of antigen-presenting cells, and the presence of anti-inflammatory cytokines that inhibit the development of an efficient T-cell activation. However, recent studies also indicate that priming and activating an adaptive anti-tumor immune response can be obtained following a patient-specific vaccination, but unfortunately, this immune response does not lead to a significant survival benefit.^24-26^ These results clearly demonstrate that peripheral T cells activation can be induced and home to the tumor, but the cold and suppressive TME of GBM is a major obstacle to overcome resistance to immune-mediated therapies. In fact, given these important difficulties, it is clear that a single agent-based therapy will be insufficient to overcome GBM resistance.

## Immunotherapy Clinical Trials in GBM

By the early 2000s, more than 1200 RCT (randomized clinical trials) have been designed and conducted worldwide clearly stating that the majority of GBM RCT have failed.^27^ In fact, despite the promising results shown by preclinical or early-phase trials, the data available to date from phase III studies have not yet confirmed a real clinical benefit. We will discuss later about completed and ongoing clinical trials of immunotherapy, analyzing both studies with immune checkpoint inhibitors, viral therapy, vaccines, and CAR T cells therapies for newly diagnosed and recurrent GBM.

### Immune Checkpoint Inhibitors

Programmed death-1 (PD-1)/PD-L1 axis and T-lymphocyte-associated protein 4 (CTLA-4) are checkpoint regulators that downregulate the immune response and can be blocked through specific antibodies enhancing T cells response against tumors. TIM3, LAG3, and TIGIT are co-inhibitory molecules and immune exhaustion markers for which some immune checkpoint inhibitors are being studied as possible treatments in several types of cancer, including GBM.

#### Anti-PD-1/PD-L1

Nivolumab is a fully human PD-1 immune checkpoint inhibitor antibody anti-PD-1 receptor. CheckMate498^28^ was an open-label phase III study in which newly diagnosed GBM patients with unmethylated MGMT (O6-methylguanine-methyltransferase) promoter were randomized 1:1 to receive RT + nivolumab vs RT + temozolomide (according to Stupp protocol).^2^ Overall, 560 patients were enrolled. The study did not meet its primary endpoint of improving overall survival (OS) with nivolumab use: mOS was 13.4 months (95% CI, 12.6-14.3) with nivolumab + RT and 14.9 months (95% CI, 13.3-16.1) with temozolomide + RT (HR 1.31; 95% CI, 1.09-1.58; *P* = 0.0037). In the same line, nivolumab was tested, in combination with temozolomide and RT, for patients with newly diagnosed MGMT promoter methylated GBM in an open-label phase III study (CheckMate548^29^). This study also did not meet its primary endpoint, demonstrating that the addition of nivolumab to concomitant chemoradiotherapy with temozolomide does not improve survival in this setting. In detail, 716 patients were enrolled and randomized 1:1 to receive nivolumab or placebo plus RT and temozolomide according to Stupp protocol. mOS was 28.9 months (95% CI, 24.4-31.6) vs 32.1 months (95% CI, 29.4-33.8), respectively (HR, 1.1; 95% CI, 0.9-1.3).

Another randomized open-label phase II/III clinical trial is ongoing (NCT 04396860) in which nivolumab and ipilimumab (anti-CTLA4) are associated with RT vs standard chemoradiotherapy with temozolomide as control arm, in newly diagnosed MGMT unmethylated GBM patients. The results of this study are not currently available.^30^

In the recurrent setting, nivolumab was evaluated in an open-label phase III trial (CheckMate 143^31^) involving 57 clinical sites in 12 countries. The study enrolled 369 patients with GBM at first recurrence following standard concomitant chemoradiotherapy and subsequent temozolomide who were randomized 1:1 to receive nivolumab 3 mg/kg or bevacizumab 10 mg/kg every 2 weeks until unacceptable adverse events, disease progression, or death. This study also failed to meet its primary endpoint of OS and secondary endpoints as 12-month OS, median PFS (mPFS), and objective response rate (ORR). Another phase II study (NCT04704154) that is active but not in the recruitment phase is evaluating the association of nivolumab with regorafenib (a multikinase inhibitor, already tested for patients with recurrent GBM^32^) in patients with recurrent GBM. Results are not available yet but are expected shortly.

Pembrolizumab is a humanized monoclonal IgG4 anti-PD-1 antibody that has been tested in patients with GBM in both phase I and phase II trials. In particular, in a phase I study^33^ 26 patients with GBM with PD-L1 expression ≥1% on tumor tissue were enrolled and treated with pembrolizumab. The results were quite disappointing with an 8% response rate. In a subsequent phase II randomized study,^34^ pembrolizumab was evaluated with or without bevacizumab in patients with recurrent GBM, also in this case the results showed a poor efficacy with an mOS of 8.8 months (combination arm) and 10.3 months (pembrolizumab alone). Pembrolizumab was also evaluated in a phase I trial^35^ in combination with hypofractionated stereotactic re-irradiation and bevacizumab in patients with recurrent GBM and anaplastic astrocytoma. The results demonstrated an mPFS of 8 months and an mOS of 13.5 months in bevacizumab naïve patients. Unfortunately, the results are difficult to interpret as the enrolled population is particularly heterogeneous. A very interesting study evaluated the use of pembrolizumab in a neoadjuvant setting in patients with recurrent GBM candidates for re-surgery^36^: 35 patients were enrolled and treated with neoadjuvant pembrolizumab, with continued adjuvant therapy following surgery (neoadjuvant arm), vs pembrolizumab given only after surgery. The results showed an mOS of 13.7 m in the neoadjuvant arm vs 7.5 m in the adjuvant arm (*P* = 0.04) with an upregulation of T-cell- and interferon-γ-related gene expression in patients receiving treatment in the neoadjuvant setting. Another study evaluating nivolumab as neoadjuvant treatment showed changes in the tumor immune microenvironment and, in particular, an enhanced expression of chemokine, immune cell infiltration and tumor-infiltrating T lymphocytes^37^; however, another similar study demonstrated that macrophages and monocytes still constitute the majority of infiltrating immune cells in GBM, even after anti-PD-1 therapy and the persistently high expression of T-cell suppressive checkpoints in these myeloid cells continues to prevent the optimal activation of T cells that infiltrate the tumor.^38^

The attempt to inhibit the CTLA-4 activity was pursued in a phase II clinical study on anti-CTLA-4 that is currently examining temozolomide treatment alone vs temozolomide with ipilimumab (anti-CTLA-4 monoclonal antibody) in patients with GBM after standard treatments.^39^ This trial is currently recruiting in 7 centers in the UK. Many other ongoing trials are evaluating the combination efficacy of anti-CTLA-4 with anti-PD-1 in treating GBM to improve the potential of these 2 therapeutic strategies that, in monotherapy, maybe have not succeeded in radically changing GBM prognosis.^40,41^

#### Anti-TIM-3, Anti-LAG-3, and Anti-TIGIT

TIM-3 is a co-inhibitory molecule expressed on immune cells^42^and its inhibition is being explored for many tumor types, including GBM, in a phase I trial in combination with anti-PD1 drugs (NCT03961971).^43^ Moreover, LAG-3 was an early marker of exhausted T cells, indicating the potential therapeutic benefit of early treatment with anti-LAG-3 drugs.^44^ Nowadays, anti-LAG-3 alone or concomitant to anti-PD1 treatment is being investigated in a phase I trial for patients with recurrent GBM (NCT02658981).^45^ Anti-TIGIT drugs directly inhibit T-cell proliferation and improve the anti-tumor immune response in many pre-clinical studies as a monotherapy or in combination with PD-1 and TIM-3 inhibitors.^46,47^ Anti-TIGIT treatment is currently in phase I clinical development for recurrent GBM in a multicenter trial in combination with antiPD-1 drugs (NCT04656535)^48^ (Table 1).

**Table 1.** Immune checkpoint inhibitors clinical trials for GBM.

Study CT identifier	Drug	Phase	Design	Patients population	No. of pts	Results mOS (months) mPFS (months) ORR (%)
NCT02617589 (CheckMate498)	Nivolumab (anti-PD-1)	III	Nivolumab + RT vs TMZ + RT	Newly diagnosed GBM MGMT unmethylated	560	mOS 13.4 vs 14.9 ORR 7.8 vs 7.2
NCT02667587 (CheckMate548)	Nivolumab (anti-PD-1)	III	TMZ + RT in combination with nivolumab or placebo	Newly diagnosed GBM MGMT methylated	716	mPFS 10.6 vs 10.3 mOS 28.9 vs 32.1
NCT02017717 (CheckMate143)	Nivolumab (anti-PD-1)	III	Nivolumab vs bevacizumab	Recurrent GBM (first recurrence)	369	mOS 9.8 vs 10 ORR 7.8
NCT04396860 (NRB-BN007 trial)	Nivolumab + ipilimumab (anti-PD-1, anti-CTLA-4)	II/III	Ipilimumab and nivolumab + RT vs TMZ + RT	Newly diagnosed GBM MGMT unmethylated	485	mPFS and mOS NA
NCT04704154	Nivolumab + regorafenib (anti-VEGF)	II	Nivolumab + regorafenib	Recurrent metastatic solid tumors	175	NA (ongoing)
NCT02054806 (Keynote 028)	Pembrolizumab (anti-PD-1)	Ib	Pembrolizumab	Recurrent GBM (any recurrence) PDL1 > 1%	26	mPFS 2.8 mOS 13.1 (mFUP 14 months) ORR 8
NCT02337491	Pembrolizumab (anti-PD-1) Bevacizumab	II	Pembrolizumab+/− bevacizumab	Recurrent GBM	80	mOS 8.8 together vs 10.3 for pembrolizumab alone ORR 20 vs 0
NCT02313272	Pembrolizumab (anti-PD-1) Bevacizumab	II	Pembrolizumab + hypofractionated stereotactic irradiation + bevacizumab	Recurrent GBM	32	mOS 13.5 mPFS 8 ORR 85 (bevacizumab naïve Pts)
Cloughesy et al.	Pembrolizumab	Randomized multi institution	Neoadjuvant and/or adjuvant therapy with pembrolizumab	Recurrent, surgically resectable GBM	35	mOS 13.7 months (neoadjuvant arm) vs 7.5 adjuvant Upregulation T cell and IFN gene expression (neoadjuvant)
ISRCTN84434175 (IpiGlioTrial)	Ipilimumab (anti-CTLA-4)	II	TMZ vs TMZ + ipilimumab after Stupp protocol	Newly diagnosed GBM	120 planned	NA
NCT03961971	MBG453 (anti TIM-3) Spartalizumab (anti-PD-1)	I	Stereotactic radiosurgery + MBG-453 + Spartalizumab	Recurrent GBM	16	NA
NCT02658981	BMS-986016 (anti LAG-3) Nivolumab	I	BMS-986016 Nivolumab	Recurrent GBM	63	NA
NCT04656535	AB154 (anti-TIGIT) AB122 (anti-PD-1)	I	AB154 + AB122	Recurrent GBM	46 planned	NA

Abbreviations: CTLA-4, cytotoxic T-lymphocyte antigen 4; GBM, glioblastoma; LAG-3, lymphocyte activation gene-3; mFUP, median follow up; mOS, median overall survival; mPFS, median progression-free survival; NA, not available; ORR, objective response rate; PD-1, programmed cell death 1; Pts, patients; RT, radiotherapy; TIM-3, T-cell immunoglobulin and mucin domain 3; TMZ, temozolomide.

### Oncolytic Viruses

Oncolytic viruses (OVs) have the antineoplastic role of selectively infecting and killing cancer cells, leaving normal cells intact by their low pathogenic effect.^49^ Moreover, OVs potentially can switch the tumor microenvironment (TME) from immunosuppressive to immunocompetent. Indeed, OV releases tumor antigens and can be armed with a transgene. There are several clinical trials that have evaluated viral therapy in patients with GBM.^50,51^

#### DNX-2401

DNX-2401 is an engineered replicative oncolytic adenovirus that contains two stable genetic changes in the adenovirus genome to selectively and efficiently infect and replicate retinoblastoma pathway deficient such as tumor cells. Preclinical studies have demonstrated DNX-2401 to be effective in glioma xenograft mouse models receiving intratumoral injections of the virus by direct oncolysis in addition to eliciting antitumor immune responses.^52,53^ In a phase I (NCT00805376) trial, 37 patients with recurrent GBM were divided into two groups: (i) patients in the first group (*n* = 25) receiving an intratumoral injection of the virus through a biopsy needle into the tumor to evaluate safety and (ii) patients in the second group (*n* = 11) receiving an intratumoral injection through an implanted catheter. In the first group, 72% (18/25) of patients showed tumor objective response. The mOS was 9.5 and 13 months for first and second group of patients, respectively.^54^ Due to the results of a phase Ib trial (NCT02197169; TARGET-I), we could assume that IFN γ did not appear to provide an additional benefit or improve survival rates compared to treatment with DNX-2401 alone.^55^ On the contrary, positive trial evidence that the concomitant administration of TMZ seems to be safe in a phase I analyzing patients with GBM at first recurrence. A phase II trial (NCT02798406: CAPTIVE/KEYNOTE-192) is evaluating the combination of DNX2401 with the anti-PD-1 antibody pembrolizumab in patients with recurrent GBM or gliosarcoma. The interim analysis (*n* = 42 patients) showed an mOS of 12.3 months.^56,57^

#### Oncolytic Polio/Rhinovirus Recombinant

The second OV that received a breakthrough therapy designation for recurrent GBM from the FDA was PVSRIPO, a genetically engineered version of the live-attenuated Sabin type 1 poliovirus (PVS).^58^ Its efficacy relies on its natural tropism to the poliovirus receptor CD155, which was found to be upregulated in GBM.^59^ NCT01491893 is a phase I study with a dose-escalation phase and subsequent dose-expansion phase, whose overall results confirmed the absence of neurovirulence although the mOS did not radically differ from historical data (12.5 months); however, the survival rate of the patients who received the experimental therapy was higher than the survival rate of the historical control group at 24 and 36 months.^60^ Therefore, it was designed a phase II trial to further evaluate PVSRIPO in this setting (NCT02986178).^61^

#### Vocimagene Amiretrorepvec (Toca 511)

Vocimagene Amiretrorepvec (Toca 511) is a γ retroviral replicating vector which encodes a gene for an optimized cytosine deaminase, capable of converting 5-fluorocytosine (5-FC) to 5-fluorouracil (5-FU).^62^ Although the results of the phase I/II studies demonstrated a good safety profile and encouraging efficacy data with durable benefit in terms of OS and some complete radiological responses with Toca 511 in patients with grade 3 gliomas and GBM^63^ this benefit was not confirmed in the subsequent phase II/III trial published by Cloughesy et al in 2020.^62^ In this randomized, open-label, phase II/III trial, 403 patients with first or second recurrence of anaplastic astrocytoma (grade 3 WHO) or GBM were randomized 1:1 to receive Toca 511 as experimental arm vs SoC (temozolomide or lomustine or bevacizumab). In Toca 511 group, Vocimagene Amiretrorepvec was administered as a local injection into the resection cavity at the time of resection and, subsequently, an oral pro-drug 5-fluorocytosine formulation (Toca-FC) was started 6 weeks after surgery, repeated every 6 weeks, at the dose of 220mg/kg/d. The study did not meet its primary endpoint of OS. The mOS was 11.1 months in the Toca 511/Toca FC arm and 12.22 months in the SoC arm (HR 1.06; 95%Ci 0.83-1.35; *P* = 0.62). No significant differences were demonstrated between the two treatment arms for the secondary endpoints (safety, durable response rate [DRR], 12-month OS, PFS and patients reported outcome, and quality-of-life analysis).

#### Sitimagene Ceradenovec

Sitimagene Ceradenovec is a replicant-deficient adenovirus that contains the cDNA for prodrug-converting enzyme, Herpes-Simplex-Virus thymidine kinase (HSV-tk). The ASPECT study was a randomized, open-label phase III trial in which patients with newly diagnosed GBM were randomized 1:1 to receive surgical resection and intraoperative perilesional injection of Sitimagene Ceradenovec followed by ganciclovir in addition to standard care vs surgical resection and standard care alone. Two hundred and fifty patients were randomized and the study met its primary endpoint: the median time to death or re-surgery was longer in the Sitimagene Ceradenovec arm (308 days, 95% CI 283-373) vs in control arm (268 days, 210-313) (HR 1.53, 95%CI 1.13-2.07; *P* = 0.006). However, the mOS was comparable in the 2 treatment arms^64^ (Table 2).

**Table 2.** Oncolytic viral therapy clinical trials for GBM.

Study	Active treatment	Phase	Design	Patients population	No. of pts	Results mOS (months) ORR (%)
NCT00805376	DNX-2401(Delta-24-RGD adenovirus)	I	Biopsy needle vs implanted catheter IT	Recurrent GBM	37	mOS 9.5 vs 13 ORR 72 (first group)
NCT01956734	DNX-2401(Delta-24-RGD adenovirus)	I	IT or resection bed inj DNX-2401 + TMZ	Recurrent GBM	31	NA
NCT02197169 (TARGET-I)	DNX-2401(Delta-24-RGD adenovirus)	Ib	IT DNX-2401 + IFNγ vs DNX-2401 alone	Recurrent GBM or gliosarcoma	37	NA
NCT02798406 (CAPTIVE)	DNX-2401(Delta-24-RGD adenovirus)	I	DNX-2401 + pembrolizumab	Recurrent GBM or gliosarcoma	49	mOS 12.3 ORR10.4
NCT01491893	PVSRIPO (Sabin type 1 poliovirus)	I	PVSRIPO IT CED	Recurrent GBM	61	mOS 12.5
NCT02986178	PVSRIPO (Sabin type 1 poliovirus)	II	PVSRIPO IT CED	Recurrent GBM	122	NA
NCT02414165	Toca 511-Toca FC (Vocimagene Amiretrorepvec and 5-fluorocytosine)	II/III	Toca 511 resection bed inj, Toca FC oral vs SoC	Recurrent, GBM or anaplastic astrocytoma	403	mOS 11.1 vs 12.22
2004-000464-28 (ASPECT)	Sitimagene Ceradenovec Herpes simplex virus-thymidine kinase (HSV-TK)	III	Surgical resection + intraoperative perilesional inj Adv-TK followed by GCV + SoC or resection and SoC alone	Newly diagnosed GBM	250	mOS 16 vs 14
UMIN000015995	G47∆ (recombinant HSV-1)	II	IT G47∆	Residual or recurrent GBM	19	mOS 28.8
NCT01301430	Parvoryx Parvovirus H-1	I/IIa	Parvoryx IT or IV and subsequently IC (resection cavity)	Newly diagnosed or recurrent GBM	18	NA

Abbreviations: Adv-TK, Sitimagene Ceradenovec; GBM, glioblastoma; GCV, ganciclovir; IC, intracerebellary; IFN, interferon gamma; INJ, injection; IT, intratumoral; IT CED, delivered intracerebrally by convection-enhanced delivery (CED); IV, intravenously; mOS, median overall survival; NA, not available; ORR, objective response rate; Pts, patients; SoC, standard of care; TMZ, temozolomide.

### Therapeutic Vaccines

Another chapter of GBM immunotherapy consists of therapeutic vaccines. Cancers vaccines can be created with “predefined” antigens (whether patient specific or tumor type shared) or with autologous tumor cells obtained from the removal of patient’s tumor. Autologous tumor cells can then be engineered to be re-injected to patients activating endogenous antigen-presenting cells (APCs) response or can be lysed and delivered to autologous APCs (dendritic cells vaccines).^65,66^

#### SurVaxM

A synthetic survivin (ubiquitous cancer-associated antigens) vaccine (SurVaxM) has been evaluated in an early trial (NCT01250470) studying 9 patients showing its safety.^67^ Furthermore, a recent phase II trial confirmed the related safety data when used as upfront therapy in 64 patients (NCT02455557). The related survival analysis evidenced an mPFS of 11.4 months, and the mOS was 25.9 months from the first SurVaxM dose.^26^ These encouraging results led to the designation of a phase II trial (SURVIVE; NCT05163080), still ongoing.

#### Rindopepimut

The Epidermal Growth Factor receptor (EGFR) is often overexpressed in GBM and it is associated with a particularly aggressive phenotype.^68^ EGFR overexpressions, amplifications, and mutations with hyperactivation of its pathway can be found in approximately 60% of GBMs^69^; the most frequent EGFR mutation in this setting is certainly EGFRvIII.^70^ Rindopepimut (CDX-110) is an EGFRvIII-targeted peptide vaccine that was evaluated in a double-blind randomized phase III clinical trial in newly diagnosed GBM patients with centrally confirmed EGFRvIII expression (ACT IV trial).^71^ Seven hundred and forty-five patients were enrolled after maximal safe surgical resection and completion of concomitant chemoradiotherapy without progression and randomized 1:1 to receive Rindopepimut or KLH (Keyhole Limpet Hemocyanin) in combination with maintenance temozolomide. The primary endpoint was OS in patients with minimal residual disease (MRD defined as tumor enhancing < 2 cm^2^ post concomitant radiochemotherapy), but the study was closed for futility following the pre-planned interim analysis. The mOS was not different in the two treatment groups with an mOS of 20.1 months (95% CI 18.5-22.1) for the Rindopepimut arm vs 20.0 months (95% CI 18 1-21 9) for the control arm (HR 1 01, 95% CI 0 79-1 30; *P* = 0 93). The mPFS was also not different between treatment arms (HR 1.01, 95% CI 0.80-1.29; *P* = 0.91) with ORR in evaluable population of 15% (95% CI 10-21) in both treatment arms. Despite the disappointing results, the same study demonstrated that Rindopepimut was able to elicit a robust humoral response in treated patients that corticosteroid use did not impact. Study treatment was well tolerated no significant differences in patient-reported quality-of-life outcomes. The most frequent serious adverse events were seizures (5%) and cerebral edema (2%).

#### IMA950

IMA950 is a multi-peptide vaccine composed of 9 MHC class I restricted peptides and 2 MHC class II-restricted peptides, c-Met and survivin, all of them overexpressed in GBM cells.^72,73^ The safety and immunogenicity of IMA950 with adjuvant poly-ICLC were assessed in a phase I/II trial (NCT01920191) in 16 patients, with an mOS of 19 months ^73^. Notably, 4 patients experienced short-term cerebral edema. Thus, it was designed another phase I/II trial to study the safety of IMA950 and poly-ICLC combined with pembrolizumab (NCT03665545).^73^

#### VXM01

VXM01 is a DNA plasmid vaccine that contains an attenuated strain of Salmonella typhimurium, which encodes the murine vascular endothelial growth factor receptor 2 (VEGFR-2) whose activation enhances angiogenesis and cell proliferation and is commonly expressed within the tumor microenvironment.^74^ NCT02718443 showed a favorable response in 5 patients with recurrent GBM. Interestingly, the prolonged survivors had lower intratumoral PD-L1 expression thus favoring the eventual combination with immune checkpoint inhibitors.^75^ In this regard, a phase I/II trial (NCT03750071) is evaluating VXM01 in combination with Avelumab in recurrent GBM.

#### APVAC1

APVAC1 contains a library of pre-curated and preprepared shared tumor antigens and patient-specific neoantigens.^76^ The safety and immunogenicity of APVAC1 were demonstrated in 15 patients with newly diagnosed GBM in a phase I study (NCT02149225), the survival analysis showed an mPFS of 14.2 months and an mOS of 29 months.^24^ Furthermore, another ongoing Phase I trial is evaluating a personalized vaccine (NeoVax) with pembrolizumab (NCT02287428).^77^

#### Autologous Vaccines

In the context of autologous vaccines as a possible treatment in patients with GBM, there are several early-phase studies in the literature that have evaluated the use of vaccines based on dendritic cells (DCs).^78,79^ These cells have the task of presenting antigens to naïve T cells to ensure the activation of an adaptive immune response. DCVax-L is the most studied DC vaccine to date, composed of autologous dendritic cells pulsed with autologous tumor lysate that was evaluated in a phase III study in newly diagnosed GBM patients, in combination with maintenance temozolomide after surgery and combined chemoradiotherapy.^80^ Patients were randomized 2:1 to receive DCVax-L plus temozolomide vs Placebo plus temozolomide; in case of disease progression/relapse during treatment, crossover was allowed. For the intent-to-treat (ITT) population of 331 patients, the mOS was 23.1 months (evaluated from the date of surgery). Due to the possibility of crossover, approximately 90% of all patients in the ITT population received DCVax-L during the study. Treatment was well tolerated with only 2.1% of patients reporting grade 3-4 adverse events possibly related to vaccine treatment. These data should be interpreted with caution considering the high crossover rate, the statistical design, and the absence of information regarding the stated primary endpoint of the study which was PFS.^75^ A phase III randomized open-label study (NCT04277221)^81^ is evaluating the use of Autologous Dendritic Cell/Tumor Antigen (ADCTA) immunotherapy in combination with a standard treatment (bevacizumab) in patients with first recurrence of GBM after the Stupp protocol. The primary endpoint was OS whereas the secondary endpoints were PFS, 6-month PFS, and 1- and 2-year survival rates. Another active but not currently recruiting phase II/III study (NCT03548571)^82^ is evaluating the use of dendritic cell immunotherapy against cancer stem cells in newly diagnosed IDH-wt, MGMT-promoter methylated GBM patients receiving concomitant radiochemotherapy with temozolomide as first-line treatment. The primary endpoint is PFS, whereas secondary endpoints include OS, assessment of patient-reported quality of life, immunological response by analysis of delayed type hypersensitivity reaction in skin and lymphocyte clonal analysis, and safety (Table 3).

**Table 3.** Therapeutic vaccines clinical trials for GBM.

Study	Active treatment	Phase	Design	Patients population	No. of pts	Results mPFS mOS (months) ORR (%)
NCT01250470	SurVaxM	I	SurVaxM + sargamostrim sc	Recurrent survivin positive GBM	9	No Grade 3-4 AE mPFS 4 mOS 20
NCT02455557	SurVaxM	IIa	SurVaxM + sargamostrim + montanide ISA 51 sc + TMZ	Newly diagnosed survivin positive GBM	64	mPFS 11.4 mOS 25.9
NCT05163080	SurVaxM	II	SurVaxM + TMZ	Newly diagnosed GBM	265 planned	NA (ongoing)
NCT0140479	CDX110 (Rindopepimut)	III	CDX-110 + GM-CSF or KLH (control arm)	Newly diagnosed EGFRvIII mut GBM	745	mPFS mOS 20.1 vs 20 ORR 15
NCT01920191	IMA 950 + Poly-ICLC	I/II	IMA 950 + Poly-ICLC + TMZ	Newly diagnosed GBM	16	mOS 19 4 Pts cerebral edema
NCT03665545	IMA950 + Poly-ICLC pembrolizumab	I/II	IMA 950 + Poly-ICLC + pembrolizumab	Recurrent GBM	61	mOS 12.5
NCT02718443	VXM01 (VEGFR-2 DNA vaccine)	I	VXM01 day 1, 3, 5, 7 then q4w after surgery if performed	Recurrent operable GBM	14	ORR 17 (DR = 1) VEGFR-2 specific T cell response (58%)
NCT03750071	VXM01 Avelumab	I/II	VXM01+ Avelumab	Recurrent GBM	30	ORR 10 (PR = 3) No AE
NCT02149225	APVAC1 (Pts specific neoantigens) Poly-ICLC	I	APVAC1 Poly-ICLC + GM- CSF	Newly diagnosed GBM	15	mPFS 14.2 mOS 29
NCT02287428	NeoVax Pembrolizumab	I	Cohort 1: NeoVax after standard RT Cohort 1a, 1b, 1c: NeoVax + pembrolizumab after standard RT	Newly diagnosed GBM	56 planned	NA (ongoing)
NCT00045968	DCVax-L (dendritic cells)	III	DcVax-L + TMZ vs placebo + TMZ (crossover allowed)	Newly diagnosed GBM	348	mOS 23.1 (ITT) 2.1% grade 3-4 AE
NCT04277221	ADCTA Bevacizumab (autologous dendritic cells)	I	ADCTA-SSI-G1 + bevacizumab	Recurrent GBM	118 planned	NA (ongoing)
NCT03548571	ADCTA	II/III	ADCTA + RT + TMZ vs RT + TMZ alone	Newly diagnosed MGMT methylated GBM	60 planned	NA (ongoing)

Abbreviations: AE, adverse event; APVAC1, actively personalized vaccines; DR, durable response; GBM, glioblastoma; ITT, intent-to-treat populations; KLH, keyhole limpet haemocyanin; mOS, median overall survival; NA, not available; ORR, objective response rate; Poly-ICLC, synthetic polyinosinic-polycytidylic acid (poly-IC) that is stabilized with poly-l-lysine (PLL); PR, partial response; Pts, patients; RT, radiotherapy; SurVaxM, SVN53-67/M57-KLH; TMZ, temozolomide.

### CAR T-Cell Therapies

CAR T cells are engineered synthetic receptors that function to redirect lymphocytes, usually T cells, to recognize and eliminate cells expressing a specific target antigen. Despite the impressive clinical responses in patients with hematologic malignancies following the treatment with CAR T cells, the efficacy of this type of strategy for solid tumors, including GBM, is to be defined yet. Only a few CAR candidates have been studied in humans with GBM, since the target of CAR T cells should not be expressed on healthy cells to avoid an autoimmune response against the brain.^83^ Brown et al^84^ conducted a phase I trial with IL-13 Ra2 CAR T (NCT02208362) and reported a case of transient complete response (PFS 7.5 months) in a patient with recurrent multifocal GBM reporting a marked improvement in terms of quality of life. These encouraging results led to the development of other CAR T-cell therapies targeting HER 2, EGFRvIII, and H3K27M due to their high expression in GBM and concomitant absence in normal cells.^39,85-87^ The results seem to be encouraging in terms of safety. Completion of clinical trials is required to evaluate their clinical safety and efficacy (Table 4).

**Table 4.** CAR T cells clinical trials for GBM.

Study CT identifier	Target	Phase	Patients population	Status	No. of pts	Results mPFS (months) mOS
NCT01454596	EGFRvIII	I/II	Malignant EGFRvIII + gliomas	Completed	18	mPFS 1.3 mOS 6.9
NCT02209376	EGFRvIII	I	Residual or recurrent EGFRvIII + gliomas	Completed	11	mOS 8
NCT03726515	EGFRvIII + pembrolizumab	I	Newly diagnosed MGMT unmethylated GBM	Completed	7	mPFS 5.2 mOS 11.8
NCT01109095	HER2	I	Recurrent HER2 + GBM	Completed	16	mPFS 3.5 mOS 24.5 PR = 1Pts SD = 7 PD = 8
NCT02208362	IL13Rα2	I	Recurrent or refractory HGG	Completed	92	CR = 1
NCT00730613	IL13Rα2	I	Recurrent or refractory HGG	Completed	3	mSurvival after relapse 11
NCT01082926	IL13Rα2	I	Recurrent or refractory HGG	Completed	6	mOS 19.7
NCT04003649	IL13Rα2 +/− ipilimumab	I	Recurrent GBM	Recruiting	60 (estimated)	NA
NCT04385173	B7-H3	I	Recurrent GBM	Recruiting	12 (estimated)	NA
NCT04077866	B7-H3	I/II	Recurrent GBM	Recruiting	40 (estimated)	NA
NCT04099797	GD2	I	GD2 + brain tumors	Recruiting	34 (estimated)	NA
NCT04214392	MMP-2 (clorothoxin)	I	Recurrent GBM, malignant gliomas and WHO grade 2 and 3 gliomas	Recruiting	36 (estimated)	NA

Abbreviations: B7-H3 B7, B7 homolog 3 protein; EGFRvIII, epidermal growth factor version III; GBM, glioblastoma multiforme; GD2, disialoganglioside 2; HER2, human epidermal growth factor 2; HGG, high grade gliomas; IL13Rα2, interleukin 13 receptor alpha 2; MMP-2, metalloproteinase-2; mOS, median overall survival; mPFS, median progression free survival; NA, not available; ORR, objective response rate; PR, partial response; SD, stable disease.

## Radiation and the Immune System: A Complex Interaction

Tumor cell death induced by RT is a very complex mechanism. The evidence of the (direct or indirect) DNA and vascular damage according to recent RT studies suggested the central role of the immune response in this process. Preclinical studies have shown that while high doses were required to induce tumor cells death in immunodeficient mice, a lower dose in immune-competent mice is sufficient.^88^

Several reviews attested the role of radiation in immune modulation improving the tumor immune response.^89-91^ These studies suggested that radiation could enhance immune response using stereotactic RT (high dose per fraction for one or few fractions) rather than conventional fractionated RT (1.8-2 Gy per fraction).^89,91^

Different outcomes were induced by radiation, including both enhancing and attenuating anti-tumor immune response through the release of tumor antigens, increasing the number of tumor-infiltrating lymphocytes, and also enhancing the activity of the dendritic cells. Moreover, irradiated tumor cells showed altered expressions of molecules such as FAS ligands and PD-L1 that are involved in programmed cell death and this may enhance immune checkpoint inhibitors efficacy.^90^

On the other hand, RT may also have an impact on myeloid-derived suppressor cells, increasing the amount of regulatory T cells.^91^ In addition, circulating naïve T cells are extremely radiation sensitive: it is known that while the tumor lethal radiation dose is 3 Gy, T-cell death may occur at 0.5 Gy. For this latter reason, RT would lead to lymphopenia, in fact lymphocytes circulating both in the high- and the low-dose area around the tumor dies.^92^

Yovino et al^93^ conducted a study to estimate the radiation dose received by lymphocytes passing through the radiation field of GBM patients, in order to explain the treatment-related lymphopenia. Based on the number of irradiations, dose rate, and radiation field size, the authors calculated the amount of blood irradiated, reporting that the proportion of blood exposed to 0.5 Gy or more, increased with the number of fractions, lower dose rates, and larger irradiation fields. In fact, the modeling determined that a single radiation fraction delivered 0.5 Gy to 5% of circulating cells, and after 30 fractions, 99% of circulating blood had received >0.5 Gy (target volume 8 cm treated by 60 Gy).

The same data about lymphopenia were reported by other authors, who proposed the blood as an immune-related organ at risk during RT.^94^ In fact, if brain volume that received 25 Gy was more than 40%, a significant increase in the frequency of acute severe lymphopenia (lymphocyte count < 500 cells/mL) was reported.^95^ Lymphopenia with a count inferior to 200 cell/mL after chemoradiotherapy in GBM could affect OS, identifying patients with poor prognosis.^96^ Moreover, in a study that evaluated the effects of RT on circulating lymphocyte count in patients treated with anti-PD-1 ICI, the authors reported that patients who developed RT-induced severe lymphopenia were more likely to have severe lymphopenia when ICI was initiated and that severe lymphopenia at that beginning of ICI therapy was associated with increased mortality reported in the multivariable analysis (hazard ratio, 2.1; *P* = .03).^97^

In conclusion, RT has a conflicting role in GBM immune response: on one hand, it induces immunogenic cell death that can contribute to immune response, while on the other hand, RT induces lymphopenia that was associated with poor prognosis and may attenuate the immune response to ICI. Probably, hypofractionation, reduction of RT target volume and decreasing dose to a healthy brain, could be useful to increase immune response in GBM.^98-100^

## Conclusions

Immunotherapy is clearly a revolution in the treatment of solid tumors. However, the role of immunotherapy in GBM remains a challenge because of the immunosuppressive nature of this tumor and its unique immune environment. Further studies will be needed to better understand possible strategies to overcome the mechanisms of immunosuppression. Data from clinical trials will show the future path, likely combination strategies may play a role.

## Data Availability

No new data were generated or analyzed in support of this research.
